# Gut Microbiota Signatures and Ecological Network Alterations Associated with Hemodialysis

**DOI:** 10.3390/microorganisms14081791

**Published:** 2026-08-14

**Authors:** Nisrine Souai, Oumaima Zidi, Panagiota Stathopoulou, Anis Bafoun, Oussama Souiai, Mariem Hanachi, Elias Asimakis, Ameur Cherif, Amor Mosbah, George Tsiamis, Soumaya Kouidhi

**Affiliations:** 1 Laboratory of Biotechnology and Valorisation of Bio-GeoRessources, Higher Institute of Biotechnology of Sidi Thabet, BiotechPole of Sidi Thabet, University of Manouba, Ariana 2020, Tunisia; nessrine.souai@fst.utm.tn (N.S.); oumaima.zidi@fst.utm.tn (O.Z.); ameur.cherif@uma.tn (A.C.); amor.mosbah@gmail.com (A.M.); 2 Department of Biology, Faculty of Sciences of Tunis, University of Tunis El Manar, Farhat Hachad Universitary Campus, Rommana, Tunis 1068, Tunisia; 3 Laboratory of Systems Microbiology and Applied Genomics, Department of Sustainable Agriculture, University of Patras, 2 Seferi St, 30100 Agrinio, Greece; panstath@upatras.gr (P.S.); eliasasim@gmail.com (E.A.); gtsiamis@upatras.gr (G.T.); 4 Unit of Hemodialysis, Military Training Hospital, Mont Fleury, Tunis 1008, Tunisia; bafounanis@gmail.com; 5 Laboratory of BioInformatics, BioMathematics and BioStatistics (BIMS), Institute Pasteur of Tunis, Place Pasteur, BP 74, Tunis 1002, Tunisia; souiai@gmail.com (O.S.); mariem.hanachi@gmail.com (M.H.); 6 Higher Institute of Biotechnology of Béja, University of Jendouba, Béja 9000, Tunisia

**Keywords:** hemodialysis, amplicon sequencing, gut microbiota, dysbiosis, dialysis vintage, microbial networks

## Abstract

Hemodialysis (HD) is the most widely used renal replacement therapy for patients with end-stage renal disease (ESRD) and is frequently accompanied by long-term complications that impair quality of life, including metabolic and inflammatory disturbances. Growing evidence suggests that these complications may be linked to alterations in the gut microbiota; however, microbial composition and interaction patterns in HD patients remain incompletely characterized. In this exploratory, cross-sectional study, high throughput 16S rRNA gene sequencing was used to profile the fecal microbiota of patients undergoing hemodialysis and of healthy controls. The objective was to characterize associations between hemodialysis and gut microbial composition, ecological network organization, and predicted functional potential. Comparative analyses revealed significant differences in bacterial community structure and microbial networks in the HD cohort. Both gender and dialysis vintage were associated with variation in specific taxa, including increased detection of the Synergistetes phylum, particularly among male patients and those undergoing long-term HD. Associations were also observed between clinical and demographic factors and the relative abundance of several short-chain fatty acid-associated taxa, including members of the Lachnospiraceae and Ruminococcaceae families and the genus *Bifidobacterium*. Predicted functional potential (PICRUSt2) indicated distinct microbial metabolic profiles in HD patients compared with controls, particularly in pathways related to carbohydrate, nucleotide, and amino acid metabolism, with additional variation according to dialysis vintage. Overall, these findings provide an exploratory characterization of structural, compositional, and predicted functional alterations of the gut microbiota associated with hemodialysis. Although the modest cohort size precludes definitive conclusions, the results support the rationale for larger, longitudinal studies investigating microbiota-derived biomarkers and host–microbiome interactions in ESRD.

## 1. Introduction

End-stage renal disease (ESRD) represents the final, irreversible stage of chronic kidney disease, characterized by the loss of renal function sufficient to maintain metabolic homeostasis, thereby necessitating renal replacement therapy. Beyond the loss of kidney function, ESRD is associated with a chronic pro-inflammatory state that substantially contributes to morbidity and mortality. Emerging evidence indicates that the intestinal tract plays a central role in this process, particularly through the translocation of bacteria and bacterial-derived products across a compromised gut barrier, thereby linking the gut microbiota to systemic inflammation in patients with ESRD [1,2]. In parallel, the gut microbiome has been recognized as a major source of uremic toxin production, further amplifying inflammatory responses and metabolic disturbances [3,4]. Consistently, elevated circulating endotoxin levels, commonly used as markers of bacterial translocation, have been identified as independent predictors of mortality in ESRD [5,6].

Several studies have demonstrated that ESRD is associated with significant alterations in the gut microbiota composition. Early phylogenetic microarray analyses revealed expansion of taxa such as Enterobacteriaceae, Halomonadaceae, and Moraxellaceae, alongside depletion of beneficial commensals, suggesting a state of gut dysbiosis primarily driven by uremia [7]. Functional analyses further identified enrichment of bacterial families harboring urease, uricase, indole-, and *p*-cresol-producing enzymes, coupled with depletion of butyrate-producing bacteria, highlighting potential mechanistic links between dysbiosis, toxin generation, and inflammation [8]. In addition to uremia, other factors common in ESRD, such as dietary restrictions, polypharmacy, and multiple comorbidities, are also likely contributors to gut microbial imbalance [9].

The type of dialysis modality may further influence the gut microbiota composition and host–microbe interactions. Recent studies have reported greater microbiome alterations in patients undergoing peritoneal dialysis (PD) than in those undergoing hemodialysis (HD), particularly in pediatric populations, although associations with inflammatory markers have not been consistently observed [10]. Conversely, large clinical cohorts have demonstrated a higher mortality risk among HD patients compared with those receiving PD, a difference that is not fully explained by vascular access or pre-dialysis care [11]. Patients on HD appear particularly susceptible to endotoxemia and innate immune dysfunction, conditions that may partially improve after kidney transplantation [12]. Moreover, comparative analyses of patients undergoing HD and PD have revealed distinct taxonomic and functional microbiome profiles, with patients undergoing HD showing an increased abundance of potentially pathogenic species, such as *Blautia obeum*, and reduced levels of beneficial butyrate-producing taxa, including *Faecalibacterium prausnitzii* and *Roseburia intestinalis*. These microbial shifts were accompanied by altered predicted metabolic functions and were associated with elevated C-reactive protein levels in HD patients, suggesting a link between dialysis modality, microbiota composition, and systemic inflammation [13]. Nevertheless, the mechanisms underlying these differences remain debated, and the extent to which hemodialysis itself contributes to microbiota alterations is still unclear.

Against this background, the present study aimed to investigate the impact of hemodialysis on the gut microbiota composition and interaction networks in adult patients with ESRD. Specifically, we examined whether dialysis vintage and patient gender were associated with differences in microbial structure and bacterial interactions within the HD population. We hypothesized that the hemodialysis process, through toxin removal, fluid shifts, and modulation of inflammatory status, may influence the gut microbial composition over time. By comparing the fecal microbiota profiles of HD patients with those of healthy controls and relating microbial patterns to clinical and demographic variables, this study seeks to provide a more detailed characterization of gut microbiota alterations associated with hemodialysis and to characterize gut microbiota composition and explore associations with maintenance hemodialysis, dialysis vintage and gender. To our knowledge, this study is among the first to combine microbial community structure, co-occurrence network analysis, and functional prediction to explore the impact of dialysis vintage and sex on gut microbiota alterations in patients undergoing hemodialysis.

## 2. Materials and Methods

### 2.1. Study Cohort

This study was conducted with the approval of the Bioethics Committee of the Military University Hospital of Tunis (No. 05032018) in accordance with the Declaration of Helsinki. Written informed consent was obtained from all participants.

A total of 19 patients undergoing hemodialysis (HD) and 18 healthy controls were enrolled. All patients were of Tunisian origin, aged between 24 and 75 years (10 females, 9 males), and adhered to a potassium-restricted diet as part of standard clinical management.

To investigate the influence of gender and dialysis duration, the HD cohort was stratified into subgroups: male (M, *n* = 9) versus female (F, *n* = 10), and short dialysis vintage (<2 years, “short” group, *n* = 7) versus long dialysis vintage (≥2 years, “long” group, *n* = 12) (Table S1). Stool samples were collected within one day of defecation, immediately frozen at −80 °C, and stored until DNA extraction.

The cohort size was modest (19 HD patients, 18 healthy controls); therefore, subgroup analyses by gender and dialysis vintage should be exploratory and may be underpowered to detect subtle differences. Age, smoking status, alcohol consumption, and the presence of associated metabolic conditions (including diabetes and hypertension) were also recorded but are not presented, as no statistically significant associations with microbial composition were detected, likely reflecting limited statistical power (*p*-adj > 0.05; Table S2).

### 2.2. DNA Extraction, First-Step PCR

Genomic DNA was extracted in triplicate (~25 mg per replicate) from fecal specimens using the InnuPREP DNA kit (Analytik Jena, Jena, Germany) according to the manufacturer’s instructions. The technical replicates were prepared as a laboratory quality-control step to assess extraction consistency; they were sequenced separately and their reads were merged prior to downstream analyses, so that a single biological sample per participant (*n* = 37) was used for all diversity, taxonomic, and network analyses. DNA quality and concentration were assessed using a Q5000 micro-volume UV–Vis spectrophotometer (Quawell Technology, San Jose, CA, USA), and samples were stored at −20 °C until PCR amplification. The V3–V4 region of the 16S rRNA gene was amplified using primers U341F-MiSeq (5′-CCT ACG GGR SGC AGC AG-3′) and 805R-MiSeq (5′-GAC TAC HVG GGT ATC TAA TCC-3′) [14]. PCR reactions (25 μL) contained 2.5 μL KAPA buffer (10×), 0.2 μL dNTPs (25 mM), 1 μL of each primer (10 μM), 0.12 μL KAPA Taq polymerase (5 U/μL), 1 μL template DNA, and 19.18 μL sterile deionized water. Thermal cycling conditions consisted of an initial denaturation at 95 °C for 3 min, followed by 35 cycles of 95 °C for 30 s, 53 °C for 30 s, and 72 °C for 1 min, with a final extension at 72 °C. for 5 min. PCR products were visualized on 1.5% agarose gel and purified using PEG/NaCl precipitation (2.5 M NaCl, 20% PEG 8000 *w*/*v* solution), washed with 70% ethanol, and resuspended in 15 μL sterile water.

### 2.3. Indexing PCR and Library Preparation

Purified amplicons were diluted to 10 ng/μL and subjected to a second PCR to attach Illumina adaptors and sample-specific indexes using the KAPA HiFi HotStart PCR Kit (Kapa Biosystems, Wilmington, NC, USA). Reactions (50 μL) contained 10 μL 5× KAPA HiFi buffer, 1.5 μL dNTPs (10 mM each), 5 μL each indexing primer (10 μM), 1 μL polymerase (1 U/μL), 2 μL template, and 25.5 μL water. Cycling conditions consisted of 3 min at 95 °C, followed by 8 cycles of 95 °C for 30 s, 55 °C for 30 s, 72 °C for 30 s, and 5 min final extension at 72 °C. Amplicons were purified using NucleoMag NGS Clean-up kits (Macherey-Nagel, Düren, Germany), quantified, pooled equimolarly (8 nM), and sequenced on an Illumina MiSeq platform (2 × 300 bp) by Macrogen (Seoul, Republic of Korea).

### 2.4. Bioinformatic Analysis

Raw reads were demultiplexed and adapter-trimmed using bcl2fastq2 v2.20.0 (Illumina, San Diego, CA, USA). The quality of raw FASTQ files was assessed using FastQC v0.12.1 [15]. FastQC reports were combined and plotted using MultiQC v1.35 [16] (Figure S1). Paired-end reads were merged, quality-filtered, and error-corrected using USEARCH v11 [17]. Paired-end reads were merged using the fastq_mergepairs command with a maximum of 10 mismatches between forward and reverse reads, a minimum Q20 for forward and reverse reads, a minimum merged length of 400 bp, and a maximum length of 520 bp. Merged reads were filtered using fastq_filter with a maximum expected error threshold of 1.0 (Emax = 1), retaining only high-quality sequences for downstream analysis. Unique sequences were identified using the -fastx_uniques command, and operational taxonomic units (OTUs) were clustered using the UPARSE pipeline (-cluster_otus) at a 97% sequence similarity threshold to ensure comparability with previous microbiome studies [18]. Singletons and chimeras were also removed using the UPARSE algorithm. Cross-talk artifacts were removed using the UNCROSS2 algorithm [19]. An OTU table mapping reads to OTUs and samples were generated using otutab. Low-abundance OTUs were filtered using otutab_trim, applying a 0.001 OTU frequency threshold (0.1% of total OTU abundance across samples). The OTU table was normalized with otutab_norm to 18,883 reads, matching the lowest sample depth (Sample C12; Table S3). Taxonomic assignment was performed in QIIME2 (v2021.2) using the feature-classifier (classify-consensus-blast) [20] against the SILVA 132 reference database, clustered at 99% similarity for 16S rRNA gene sequences [21,22]. Rarefaction curves for observed features and Shannon diversity were generated in QIIME 2 using the alpha-rarefaction command, with the initial OTUs table before normalization and trimming. Good’s coverage was also estimated using the initial OTUs read-count table. Relative abundance profiles at the phylum and genus levels were generated, and alpha diversity metrics were computed using the R package MicrobiomeSeqv0.1 [23]. Relative abundance and diversity metrics were calculated based on the normalized and trimmed OTUs table (OTUs table).

Overall statistical significance of alpha diversity indices was calculated using ANOVA with the Benjamini–Hochberg correction for multiple group comparisons. Pairwise comparisons between groups were performed using Tukey’s Honestly Significant Difference (HSD) test. To test for significant variation in relative abundance between the control group and the HD group the Rhea v1.1.8 pipeline was used [24]. Differential abundance analysis was also performed using multivariable linear model testing in MaAsLin3 v1.5.3 [25] with total sum-scaled (TSS) normalized, log2-transformed abundances. False discovery rates (q-values) were calculated using the Benjamini–Hochberg correction. The primary factor tested was cohort status (HD group vs. control), adjusted for HD duration, gender, diabetes, hypertension, hepatitis C, antibiotic use, alcohol consumption, tobacco use, and physical activity. Statistical significance was evaluated using the default program threshold of q ≤ 0.1. Beta diversity was explored using principal coordinate analysis (PCoA) and canonical analysis of principal coordinates (CAP). The analysis was based on Bray–Curtis similarity, and statistical significance was evaluated with PERMANOVA+ (999 permutations) [26]. Adjusted *p*-values were produced using the Bonferroni correction. Tests of homogeneity of dispersion were performed using the betadisper function in the R package vegan v2.7-1 [27]. ANOVA tests were used to test overall differences in dispersion and Tukey’s HSD for pairwise comparisons. Statistical analyses, including ANOVA, Tukey’s HSD, PERMANOVA, and tests for homogeneity of dispersion were performed with the R package vegan v2.7.2 [27]. For all statistical tests, a significance threshold of 0.05 was used.

Because of the relatively small cohort size, subgroup analyses were considered exploratory. Differential abundance analyses were therefore interpreted as hypothesis-generating rather than confirmatory, and no causal inferences were drawn from the observed associations.

### 2.5. Microbial Network and Functional Predictions

Microbial co-occurrence and association networks were constructed using the CoNet plugin in Cytoscape 3.8.1 [28]. The development of the networks was based on the example described in the results section of the CoNet publication [29]. In brief, the OTUs table and the taxonomy classifications were used as input files, and Pearson, Spearman, mutual information, Bray–Curtis, and Kullback–Leibler metrics were used as the network construction methods. The option “explore links between higher taxa” was enabled to compute correlations between higher-level taxa (e.g., genera and families, etc.). Edge significance was assessed using 1000 permutations and 1000 bootstrap distributions. The resulting *p*-values were merged with the brown method and adjusted with the Benjamini–Hochberg correction method. Nodes represent bacterial taxa and edges represent predicted relationships between nodes. Node size reflects the interaction degree. Positive correlations were interpreted as potential co-occurrence or shared ecological preferences, whereas negative correlations were interpreted as potential competitive or exclusion relationships. Network layouts were produced using Gephi v0.10 [30] and the built-in Fruchterman-Reingold algorithm [31].

Predicted functional profiles were generated with PICRUSt2 v2.6.2 using the default picrust2_pipeline.py script [32], based on the representative 16S rRNA gene sequences of each OTU and the OTUs table. The PICRUSt2-SC database [33], which is based on the Genome Taxonomy Database (GTDB) release 214 [34], was used as a phylogenetic reference database. KEGG Ortholog (KO) and Enzyme Commission (EC) gene family annotations were mapped to GTDB genomes using open-access KO and EC identifiers. KO and EC gene families were mapped to the MetaCyc database v24 [35] metabolic pathways using MinPath v1.2 [36]. The R package ggpicrust2 v2.5.17 [37] was used for statistical analysis and visualization. Pathway abundance values were normalized with CPM (counts per million) normalization. Wilcoxon rank-sum tests with the Benjamini–Hochberg correction were used for pairwise comparison of differentially abundant pathways. Heatmaps were generated using z-transformed pathway abundances (row-wise centering and scaling) with the “scale” function. The 30 most significantly differentiated pathways were included in the heatmaps (with the lowest *p*-adjusted). Differential pathway abundance was also assessed using multivariable linear models in MaAsLin3 v1.5.3 [25], following the same procedure described previously for bacterial taxa. As PICRUSt2 provides predicted and not directly measured functional profiles, interpretations were limited to inferred metabolic potential rather than confirmed functional activity.

### 2.6. Methodological Considerations

This study was exploratory and cross-sectional. 16S rRNA sequencing allows taxonomic profiling but has limited species-level resolution and functional inference compared with shotgun metagenomics. Dietary intake, medications, and lifestyle factors (e.g., smoking) were not systematically controlled. While clinical data were collected, residual confounding could not be excluded. These methodological factors should be considered when interpreting microbial patterns and interaction networks.

## 3. Results

### 3.1. Cohort Characteristics and Sequencing Output

Fecal samples were collected from 19 hemodialysis patients (10 women and 9 men) and 18 healthy controls. Patients with HD were further stratified by dialysis vintage into short (<2 years, *n* = 7) and long (≥2 years, *n* = 12) subgroups. High-throughput sequencing generated 2,066,603 high-quality filtered reads, corresponding to an average of 55,854 reads per sample and a total sequencing yield of approximately 0.96 Gbp (Table S3). Rarefaction curves based on the Shannon index saturated at approximately ten thousand reads while observed feature curves were nearly plateaued at approximately fifteen to twenty thousand reads (Figure S2). Additionally, Good’s coverage was calculated at 0.990–0.999 for all the samples (Table S3). The above indicates that the samples had reached sufficient saturation at the minimum normalization depth of 18,883 reads. Clinical, demographic data are presented in Table S1, while alpha diversity metrics are presented in Table S4. The reported results are based on the analysis of 16S rRNA gene data obtained from across all groups. Five main bacterial phyla were detected: Bacteroidetes, Firmicutes, Proteobacteria, Verrucomicrobia and Actinobacteria. At the genus level, *Prevotella* 9 and *Faecalibacterium* were the most abundant taxa.

### 3.2. Gut Microbiota Diversity Metrics

**Figure 1:**
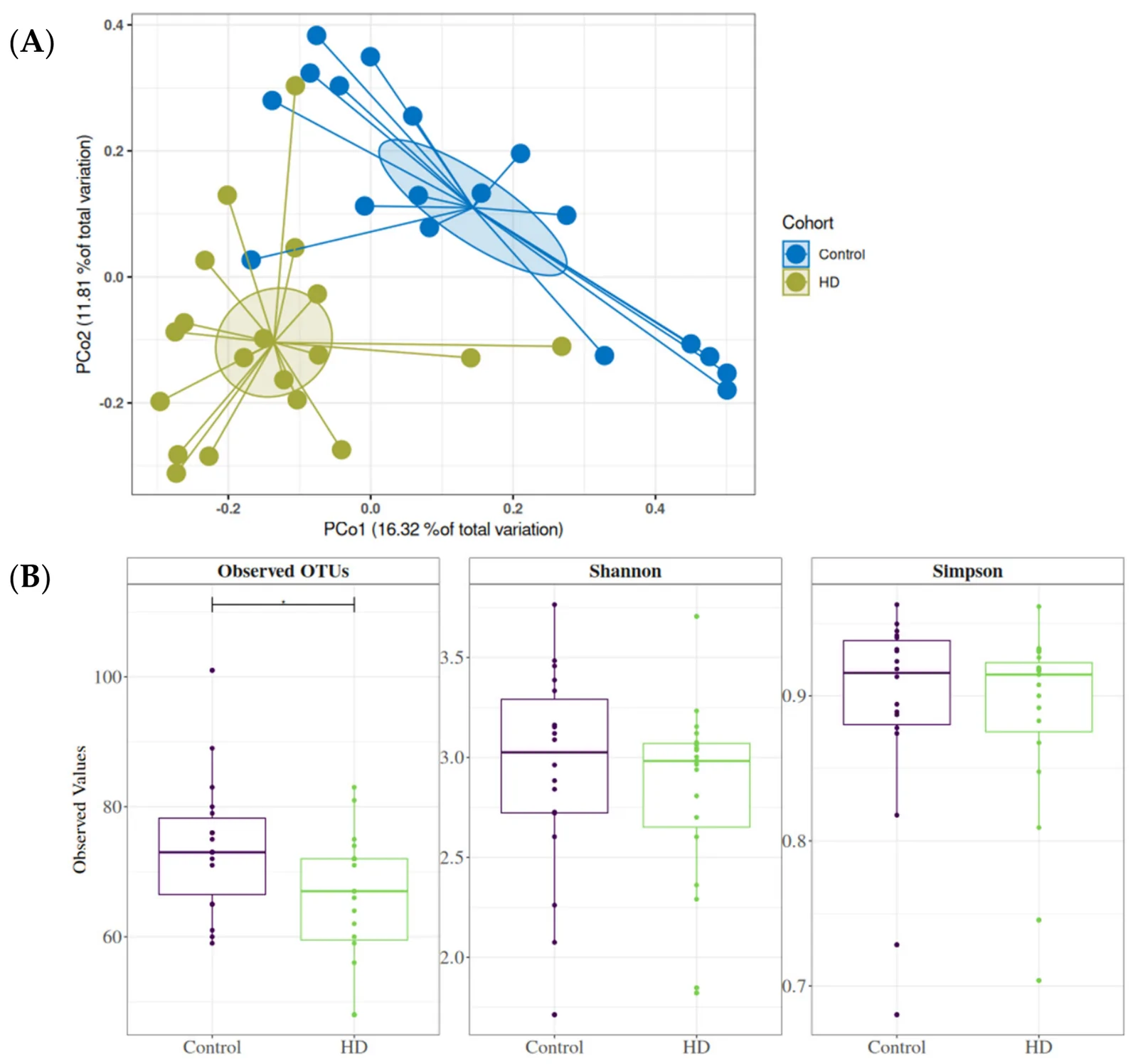
Overall gut microbiota diversity in hemodialysis patients and controls (**A**) Principal coordinate analysis (PCoA) based on Bray–Curtis distances showing separation between hemodialysis (HD) patients (*n* = 19) and healthy controls (*n* = 18). (**B**) Alpha diversity comparison between patients with HD and controls based on observed OTUs (richness). Boxes represent interquartile ranges, horizontal lines indicate medians, and whiskers denote the minimum and maximum values. Overall differences were assessed by ANOVA with Benjamini–Hochberg correction, and pairwise comparisons by Tukey’s HSD test. * Statistically significant difference between study groups.

Alpha diversity was evaluated using the Observed OTU, Shannon, and Simpson diversity indices (Figure 1B, Table S4). Observed OTU richness was significantly lower in HD patients than in healthy controls (*p* = 0.0226). In contrast, the Shannon and Simpson indices did not differ significantly between groups (adjusted *p* > 0.05), indicating that hemodialysis was associated with reduced richness rather than a change in overall community evenness. Exact and adjusted *p*-values are reported in Table S2.

Beta diversity analysis using analysis of principal coordinates (PCoA) revealed a clear separation between patients with HD and healthy controls (*p* = 0.001, Figure 1A). PERMANOVA confirmed that overall microbial community composition differed significantly between groups (*p* < 0.05), indicating compositional restructuring associated with hemodialysis.

### 3.3. Taxonomic Composition

**Figure 2:**
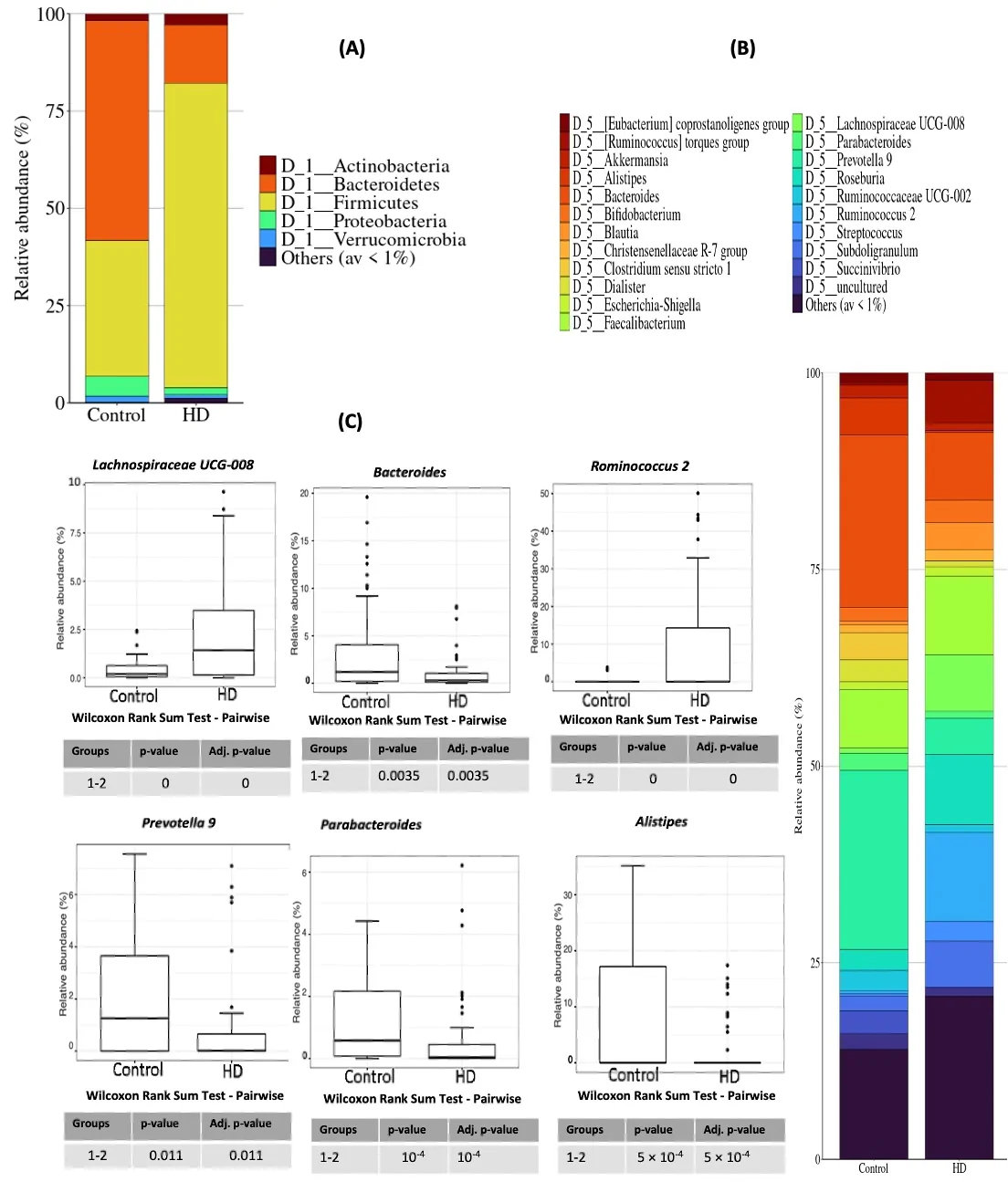
Taxonomic composition of gut microbiota in hemodialysis patients (**A**) Mean relative abundance of dominant bacterial phyla in healthy controls and HD patients. (**B**) Mean relative abundance of dominant bacterial genera (>1%) in the control and HD groups. (**C**) Boxplots of genera identified as significantly different between patients with HD and controls by serial group comparison analysis (Wilcoxon rank-sum test with multiple-testing correction).

At the phylum level, HD patients showed a higher relative abundance of Firmicutes (78.25% vs. 34.88%) and lower abundance of Bacteroidetes (14.97% vs. 56.55%) than the controls (*p* < 0.0001). Proteobacteria were also reduced in HD patients (1.77% vs. 5.07%, *p* = 0.0034), whereas Synergistetes appeared exclusively in HD samples (1.03%, *p* = 0.05; Figure 2A).

At the genus level, SCFA-producing genera, including *Bifidobacterium*, *Faecalibacterium*, and *Ruminococcus*, were enriched in HD patients, whereas *Prevotella* 9 and *Bacteroides* were decreased (Figure 2B, Table 1). Serial group comparison identified six genera with significant differences between HD patients and controls (Lachnospiraceae UCG-008, Parabacteroides, Alistipes, Bacteroides, *Prevotella* 9, and *Ruminococcus* 2; Figure 2C). Evaluation of differential abundance between hemodialysis patients (HD) and control individuals using multivariable analysis adjusted for clinical and demographic factors did not yield significant results after False Discovery Rate correction (q > 0.1) (Table S5). This result could be attributed to the small sample size and the large number of covariates that were included in the model. Nonetheless, some genera showed differences between groups based on the uncorrected *p*-value (*p* ≤ 0.05) (Table S5, Figure S3), with taxa belonging to Ruminococcaceae (1.8–0.13% average relative abundance), *Odoribacter* (0.27%), and *Citrobacter* (0.25%) exhibiting the strongest variation. Rare genera (≤0.6% average relative abundance), such as *Holdemanella*, *Citrobacter*, and *Erysipelatoclostridium*, showed positive association with HD patients, while the [*Eubacterium*] *hallii* group and *Odoribacter* were negatively associated. Additional variation in the relative abundance of bacterial genera was linked to clinical and demographic covariates, as shown in the heat map (*p* ≤ 0.05) (Table S5, Figure S3).

### 3.4. Microbial Interaction Networks

**Figure 3:**
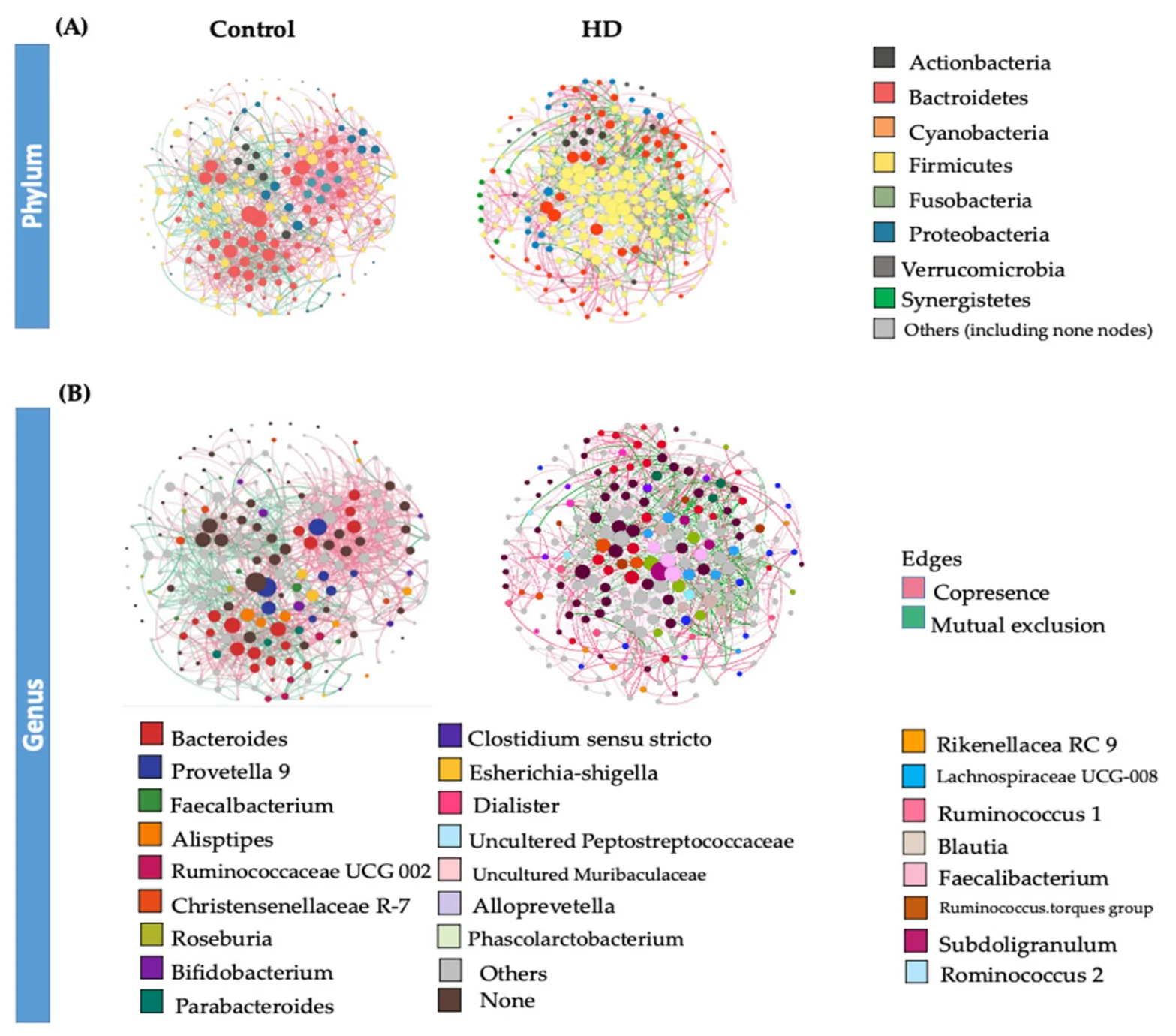
Microbial interaction networks in healthy controls and hemodialysis patients Co-occurrence and mutual exclusion networks constructed from fecal microbiota of (**A**) 18 healthy controls and (**B**) 19 HD patients. Nodes represent operational taxonomic units (OTUs) colored by phylum affiliation, and edges represent significant positive (co-occurrence) or negative (mutual exclusion) associations (*p* < 0.05). Node size is proportional to the number of connections.

Co-occurrence network analysis indicated that patients with HD exhibited less interactions (edges = 898) and a lower clustering coefficient (0.201) than control individuals (edges = 1273, clustering = 0.245), suggesting altered network structure and connectivity (Figure 3, Table 2). Hub genera in HD patients included *Faecalibacterium* and *Subdoligranulum*, whereas controls were dominated by *Bacteroides* and *Prevotella* 9. In contrast, healthy control networks displayed: greater modularity, higher clustering coefficients and a higher percentage of negative interactions. These structural differences indicate that hemodialysis is associated not only with compositional shifts but also with reorganization of ecological interaction networks, potentially reflecting decreased resilience and functional redundancy.

### 3.5. Influence of Gender

Alpha diversity analysis was first evaluated in a sex-stratified manner by comparing female HD patients with female healthy controls and male HD patients with male healthy controls, revealing significant differences between groups (Table S4, Figure S4). This approach was used to account for the known influence of sex on gut microbiota composition. However, due to the limited sample size after stratification, detailed taxonomic, beta diversity, and network analyses were primarily conducted through pairwise comparisons between male and female HD patients to explore sex-associated microbial patterns within the disease cohort.

**Figure 4:**
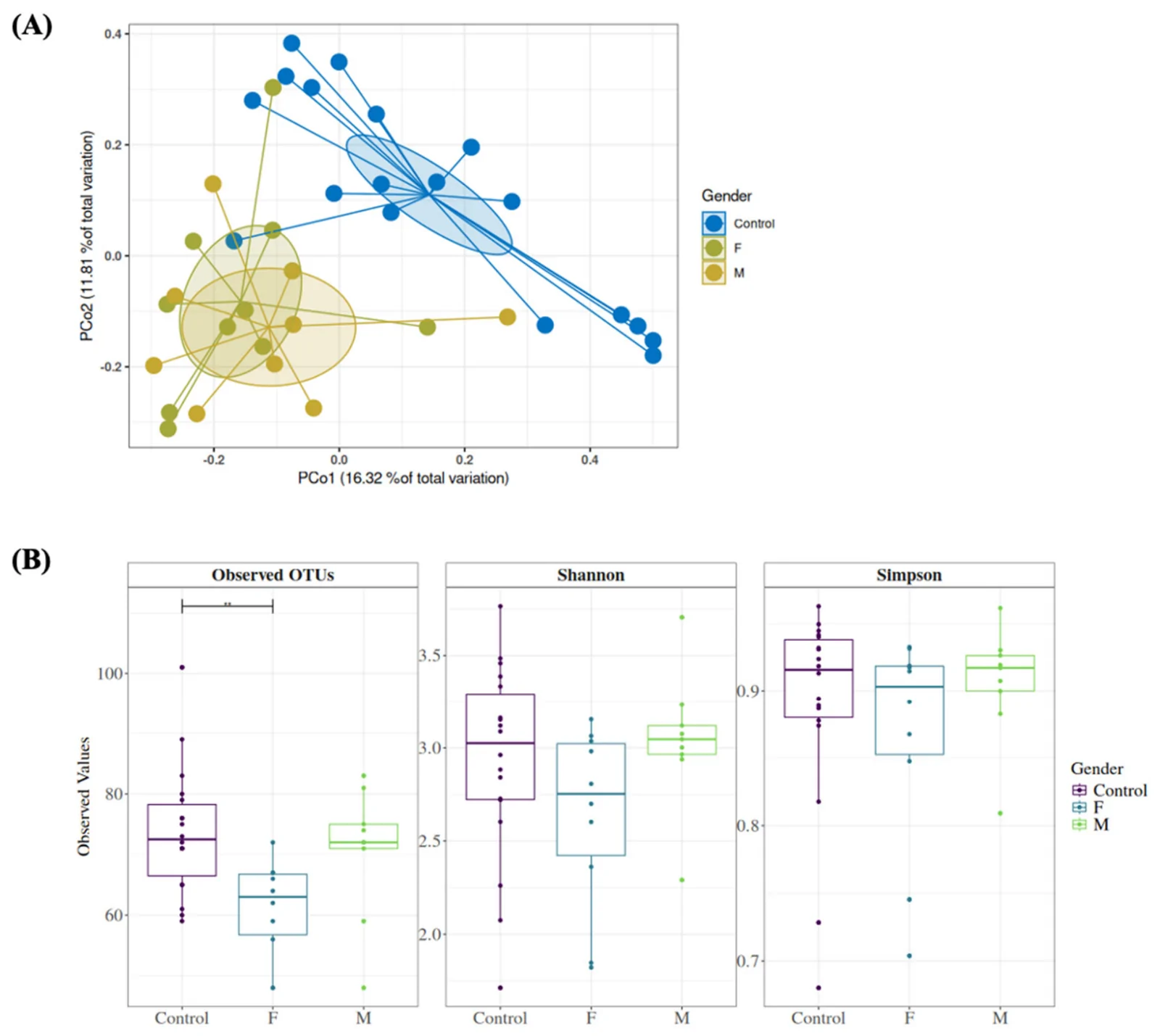

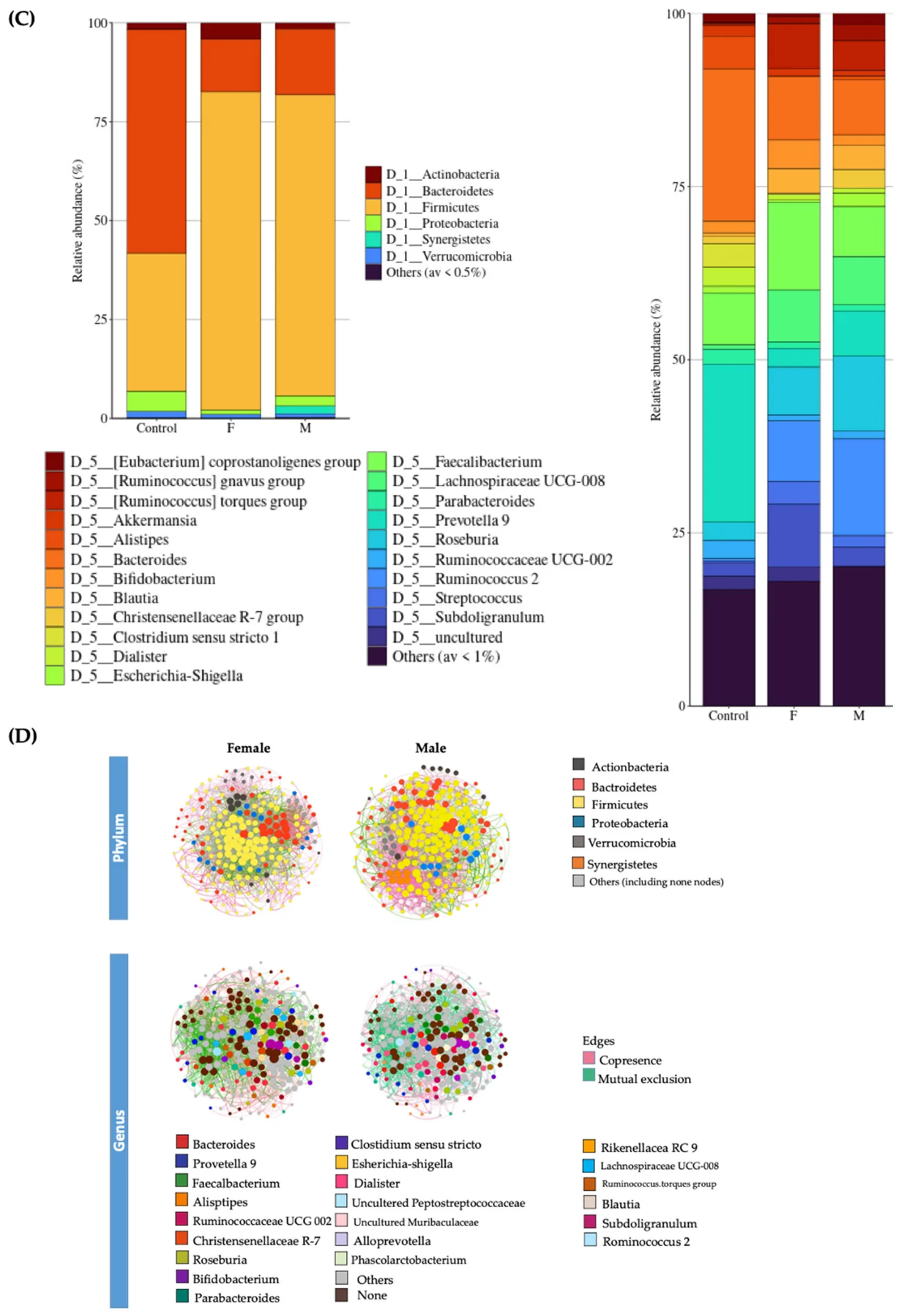
Effect of gender on gut microbiota structure in hemodialysis patients. (**A**) PCoA analysis showing separation between male (*n* = 9) and female (*n* = 10) HD patients and 18 controls. (**B**) Alpha diversity (Shannon index) comparison between male and female patients with HD. (**C**) Relative abundance of selected bacterial phyla and genera showing significant differences between genders. (**D**) Co-occurrence network analysis of gut microbiota in male and female HD patients. Nodes represent OTUs colored by phylum, and edges indicate significant microbial associations (*p* < 0.05). ** High statistically significant difference.

Alpha diversity (Shannon index) was lower in females than in males (*p* = 0.01; Figure 4B). CAP analysis revealed a separation between HD men and women (*p* = 0.001; Figure 4A). At the phylum level, Actinobacteria were more abundant in HD women (4.14% vs. 1.5%, *p* = 0.0107), and Proteobacteria and Synergistetes were more abundant in HD men (Proteobacteria: 2.49% vs. 1.05%, *p* = 0.0105; Synergistetes: 2.07%, vs. 0% *p* = 0.0072). Notably, Synergistetes abundance was higher in male HD patients, suggesting a potential association between dialysis vintage, gender, and enrichment of specific anaerobic taxa. At the genus level, *Faecalibacterium* and *Subdoligranulum* were reduced in HD men, whereas *Prevotella* 9 was increased (Figure 4C). In general, gender-based comparisons indicated distinct microbial patterns between male and female HD patients, although these findings should be interpreted cautiously due to limited sample size.

Network analysis showed higher connectivity in men (edges = 2873, clustering = 0.153) than in women (edges = 2081, clustering = 0.130; Figure 4D, Table S6). It is important to note that co-occurrence networks represent statistical associations based on abundance patterns and do not imply direct ecological or causal interactions between taxa.

### 3.6. Influence of Dialysis Vintage

**Figure 5:**
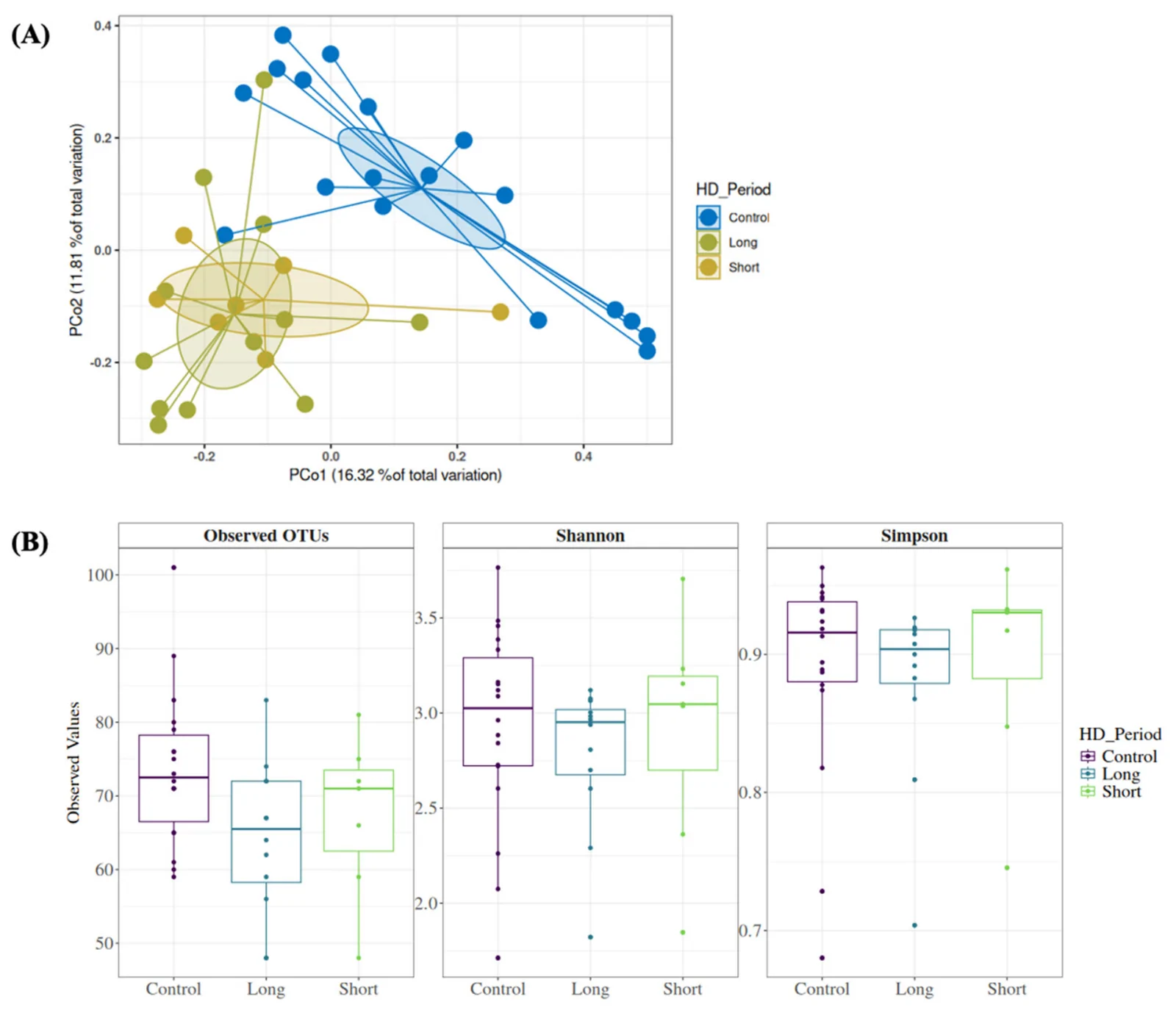

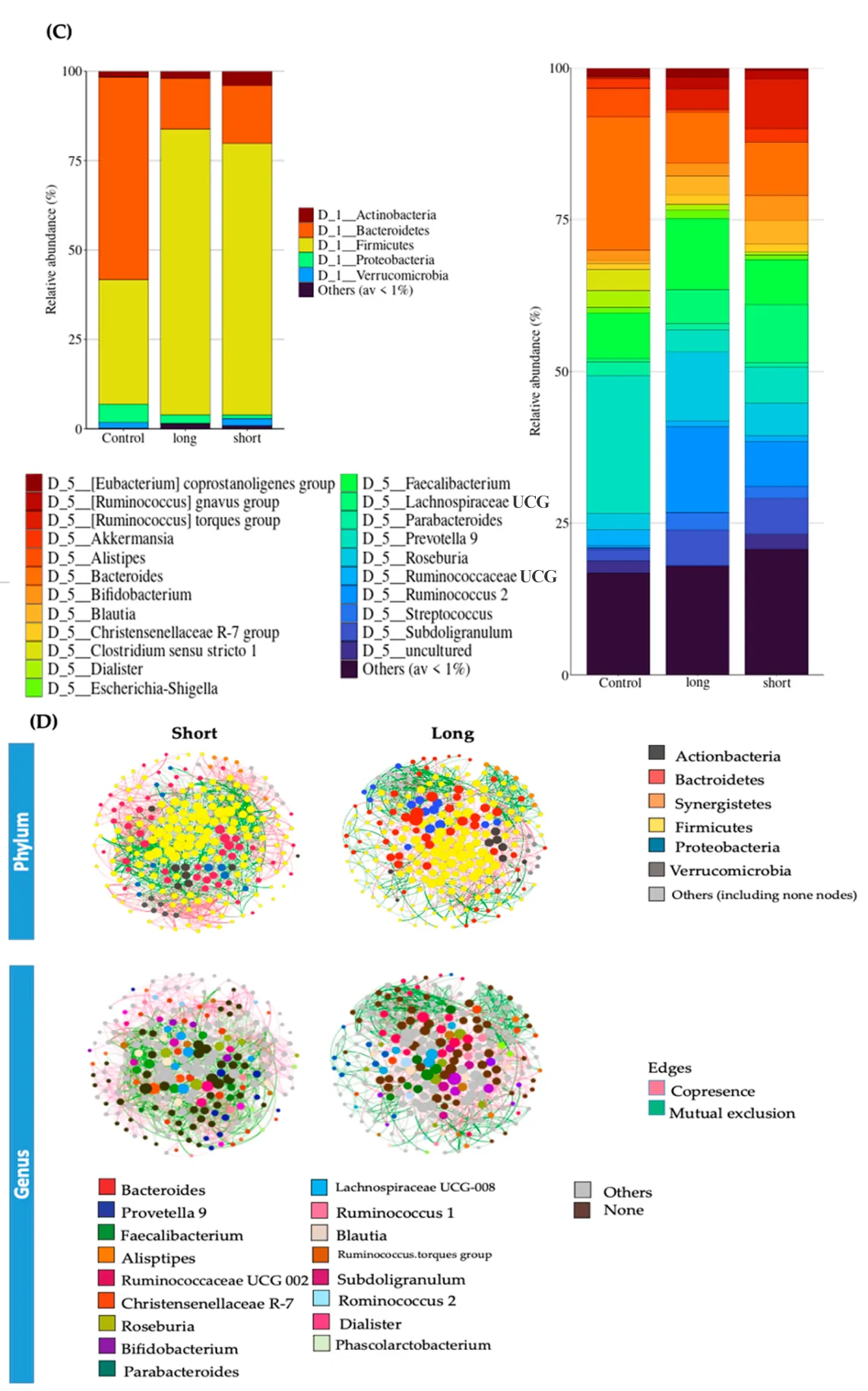
Effect of dialysis vintage on gut microbiota composition and interactions (**A**) PCoA analysis comparing HD patients with short (<2 years; *n* = 7) and long (≥2 years; *n* = 12) dialysis vintage and 18 healthy controls. (**B**) Alpha diversity (Observed OTUs) across the dialysis vintage subgroups. (**C**) Relative abundance of selected bacterial phyla and genera that differed between short- and long-term HD patients. (**D**) Co-occurrence networks of gut microbiota in short- and long-term HD patients. Nodes represent OTUs colored by phylum, and edges indicate significant associations (*p* < 0.05).

PCoA analysis indicated a slight separation between short- and long-term HD patients (*p* = 0.043; Figure 5A). Alpha diversity (Observed OTUs) was lower in patients undergoing long-term HD than in controls (*p* = 0.0087; Figure 5B). Meanwhile, there was no significant difference between short and long subgroups (*p* = 0.07).

Phylum-level analysis revealed decreased Verrucomicrobia (0.22% vs. 2.03%, *p* = 0.001) and increased Proteobacteria (2.31% vs. 1%, *p* = 0.045) in patients undergoing long-term HD. At the genus level, SCFA-producing bacteria, such as *Bifidobacterium*, *Ruminococcus* *torques* group, and *Lachnospiraceae* UCG-008, were enriched in short-term HD patients, whereas *Roseburia*, *Ruminococcus* 2, and *Faecalibacterium* were more abundant in long-term patients (Figure 5C, Table 3).

Network analysis showed fewer edges and lower clustering in long-term HD patients (edges = 1307, clustering = 0.169) compared to short-term HD patients (edges = 1904, clustering = 0.203), with hub genera shifting from *Dialister* and *Alistipes* in short-term to *Lachnospiraceae* UCG-008 and *Bifidobacterium* in long-term HD (Figure 5D, Table S7).

### 3.7. Predicted Functional Profiles

**Figure 6:**
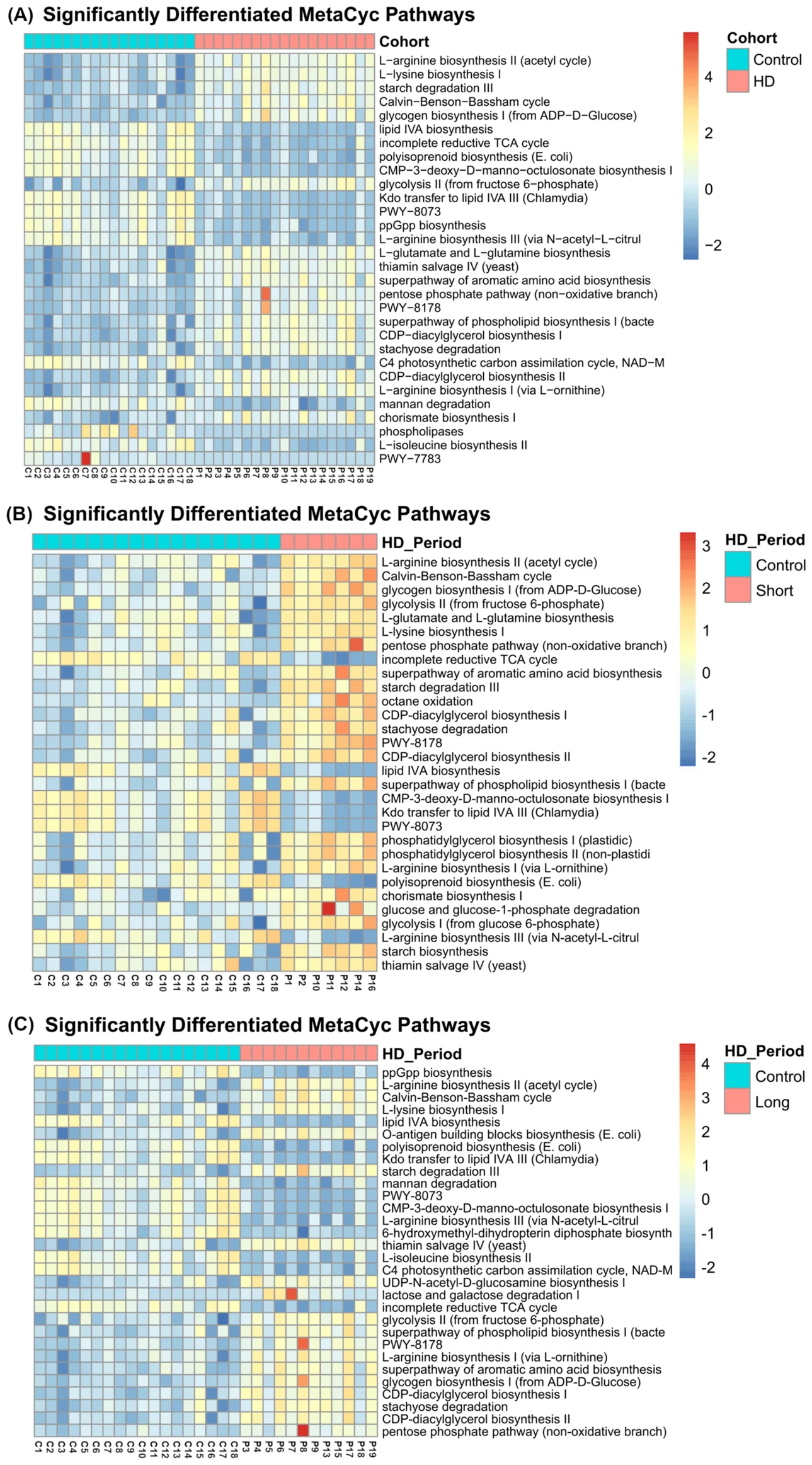
Heatmaps showing the predicted functional profiles of the gut microbiota inferred using PICRUSt2 v2.6.3. MetaCyc pathway abundances were normalized using counts per million (CPM) and visualized as row-wise z-transformed values. The 30 most significantly differentially abundant pathways (lowest Benjamini–Hochberg-adjusted *p*-values) are shown. (**A**) Comparison between patients undergoing hemodialysis (HD) and healthy controls. (**B**) Comparison of predicted functional profiles between short-term (<2 years) and healthy controls. (**C**) Comparison of predicted functional profiles between long-term (≥2 years) HD patients and healthy controls.

Predicted functional profiling of the fecal microbiota was performed using PICRUSt2 to infer MetaCyc metabolic pathways from the 16S rRNA gene data. Differential pathway abundance analysis identified significant differences in the predicted metabolic potential between hemodialysis (HD) patients and healthy controls after Benjamini–Hochberg correction. The most prominent alterations involved pathways associated with carbohydrate metabolism, amino acid biosynthesis, nucleotide metabolism, and central carbon metabolism (Figure 6A).

Compared with healthy controls, the HD microbiota showed significant enrichment of several predicted biosynthetic pathways, including L-arginine biosynthesis II (acetyl cycle), L-lysine biosynthesis I, and starch degradation III (adjusted *p* < 0.001). Additional differences were observed in pathways involved in carbon fixation and intermediary metabolism, suggesting an overall shift in the predicted metabolic capabilities of the intestinal microbial community in HD patients.

Gender-based stratification within the HD cohort indicated differences in the predicted functional potential between female and male patients. Although several pathways differed (*p*-values < 0.05) (Table S8), including pathways related to fucose and rhamnose degradation, D-galactarate degradation and NAD salvage metabolism, none remained statistically significant after correction for multiple testing (Table S8). Consequently, no robust sex-associated differences in predicted microbial function were identified in this cohort.

Dialysis vintage was associated with distinct functional profiles. Compared to healthy controls, patients undergoing short-term hemodialysis exhibit enrichment of predicted pathways involved in amino acid biosynthesis, glycogen biosynthesis, glycolysis and central carbohydrate metabolism (Figure 6B and Figure S5).

Similarly, long-term HD patients demonstrated significant alterations in predicted metabolic pathways, including increased representation of L-arginine biosynthesis II and L-lysine biosynthesis I, together with changes in ppGpp biosynthesis and other central metabolic pathways (Figure 6C and Figure S6). These findings indicate that predicted microbial metabolic potential may continue to evolve during prolonged hemodialysis.

Overall, PICRUSt2 analysis suggests that gut microbial dysbiosis in HD patients is accompanied by alterations in predicted metabolic functions, particularly those involved in carbohydrate utilization, amino acid biosynthesis, and nucleotide metabolism. Because PICRUSt2 results are based on computational inference from 16S rRNA gene profiles rather than direct metagenomic, transcriptomic or metabolomic measurements, they should be interpreted as predicted functional potential rather than confirmed metabolic activity.

Multivariate analysis of PICRUSt2-inferred pathways using MaAsLin3 identified a set of predicted metabolic functions whose abundances were significantly associated with clinical and lifestyle covariates (q ≤ 0.1). Antibiotic exposure (ATB yes) produced the strongest signal across the dataset, showing positive associations (β coefficient = 0.01–0.26) with a broad range of amino-acid and nucleotide biosynthesis pathways (Figure S7, Table S9). Predicted pathways such as L-methionine biosynthesis IV (PWY-7977), L-lysine biosynthesis III (PWY-2942), 5-aminoimidazole ribonucleotide biosynthesis I (PWY-6121), and several additional lysine, threonine, valine, and purine biosynthesis routes displayed significant associations (q ≤ 0.1), suggesting that antibiotic exposure may influence the predicted functional potential of the microbiota.

Beyond antibiotic use, the analysis revealed secondary associations with other covariates. Hemodialysis (HD) patients showed predicted negative association with one pathway (5-aminoimidazole ribonucleotide biosynthesis I; PWY-6121) compared to the control group. Alcohol consumption (Alcohol yes) was the second most influential covariate and was linked to increased predicted abundance in several nucleotide and amino-acid biosynthesis pathways. Additional associations reached significance at the uncorrected *p*-value level (*p* ≤ 0.05) but did not retain their significance after FDR-adjustment (Table S9). Patients with hepatitis C (Hepatitis_C yes) showed negative associations with six pathways, most prominently the superpathway of L-alanine biosynthesis (PWY0-1061), which exhibited a large negative coefficient (−1.15). Gender (F) and Diabetes (yes) each displayed few but strong effects (q ≤ 0.1). Female individuals were associated with decreased abundance of lipid IVA and related biosynthesis pathways (β ≈ −4), while diabetes showed a strong positive association (β = 7.9) for L-lysine fermentation to acetate and butanoate (P163-PWY) (Figure S7, Table S9). Other covariates, including HTA, Tobacco, Sport, and HD duration, showed scattered associations, most of which did not pass FDR correction.

Overall, the multivariate analysis results indicate that antibiotic exposure may act as a primary driver of predicted functional shifts, with increases across multiple biosynthetic pathways. Alcohol use, hepatitis C, gender, and diabetes may exert additional but comparatively smaller effects.

## 4. Discussion

The present study investigated the gut microbiota composition, diversity, and inferred functional potential in patients with end-stage renal disease (ESRD) undergoing hemodialysis, with a particular focus on the associations with dialysis vintage and patient gender. Using 16S rRNA gene sequencing and complementary ecological analyses, we identified marked differences in microbial structure and interaction patterns between patients undergoing hemodialysis and healthy controls. These differences were further modulated by dialysis duration and sex, suggesting that both clinical and demographic factors are associated with variations in the gut microbiota in this population. These findings highlight the association between hemodialysis-related clinical factors and gut microbiota alterations, rather than implying direct causal relationships. These patterns invite confirmation in larger longitudinal cohorts, signaling scientific rigor while sustaining optimism for future research avenues.

Consistent with previous studies in chronic kidney disease (CKD), we observed a reduction in bacterial richness in patients undergoing hemodialysis compared with healthy controls, supporting the concept that end-stage renal disease and renal replacement therapy are associated with gut microbial dysbiosis [38,39,40,41,42]. Reduced alpha diversity has been widely associated with impaired ecosystem resilience and adverse health outcomes across multiple chronic conditions, including metabolic and inflammatory diseases [43,44,45], and has also been reported in CKD populations [38,39,40,41,42]. In our cohort, bacterial richness was further reduced in patients with a longer dialysis vintage, suggesting a cumulative association between prolonged hemodialysis exposure and progressive microbiota alterations. Similar reductions in richness have been reported by Luo et al. [38], who observed decreased Chao1 indices in dialysis patients compared with controls, although without significant differences in Shannon diversity. Shannon diversity in our study showed a trend toward lower values in women undergoing hemodialysis, suggesting that sex may influence microbiota composition, although this observation requires further validation.

At the taxonomic level, patients undergoing hemodialysis exhibited an increased relative abundance of Firmicutes and a reduction in Bacteroidetes, consistent with patterns previously reported in CKD cohorts [38,39,40]. These phylum-level shifts may be relevant given the role of the gut microbiota in nutrient metabolism, immune regulation, and inflammatory signaling [46]. However, such changes should be interpreted cautiously, as the Firmicutes-to-Bacteroidetes ratio is influenced by multiple host and environmental factors and does not necessarily imply functional consequences. Importantly, these taxonomic associations should not be interpreted as independent effects of hemodialysis itself, since residual confounding by diabetes, inflammation, medication use, dietary habits, and other clinical variables cannot be excluded in the present cross-sectional study.

Within Firmicutes, several genera commonly associated with short-chain fatty acid (SCFA) production, including Faecalibacterium, Roseburia, and members of the Lachnospiraceae and Ruminococcaceae families, showed variation in relative abundance according to dialysis vintage. Patients with shorter dialysis duration exhibited relatively higher abundances of these taxa, whereas longer dialysis vintage was associated with increased representation of other Firmicutes members, including *Ruminococcus*. Previous studies have linked *Ruminococcus* species to CKD and inflammatory or metabolic conditions [47], although their functional roles are likely strain-specific and context-dependent. These findings suggest a potential restructuring of SCFA-associated microbial niches with prolonged dialysis exposure, although SCFA production was not directly measured in this study.

Interestingly, the relative enrichment of *Faecalibacterium* and *Bifidobacterium* observed in our hemodialysis cohort contrasts with many studies reporting depletion of these beneficial taxa in CKD. However, this pattern is not universal. For example, Hu et al. (2020) also identified *Faecalibacterium* and members of the *Bifidobacteriaceae* among the dominant taxa in CKD and hemodialysis patients, highlighting the heterogeneity of gut microbiota profiles across cohorts [48]. These discrepancies may reflect differences in geography, diet, medication use, dialysis practices, and sequencing methodologies. Moreover, genus-level abundance does not necessarily reflect species composition or functional activity. Therefore, our findings should be interpreted cautiously and warrant validation in larger, multicenter studies integrating functional analyses.

In contrast, several genera within the Bacteroidetes phylum, including Prevotella, Parabacteroides, and Bacteroides, were less abundant in hemodialysis patients compared with controls, consistent with previous reports in CKD [39,40]. These taxa contribute to carbohydrate fermentation and SCFA production, and their depletion may influence metabolic interactions within the gut ecosystem. However, functional consequences cannot be directly inferred from taxonomic data alone.

A notable observation in this study was the detection of the phylum Synergistetes exclusively in hemodialysis patients in our dataset, particularly among men and individuals with longer dialysis vintage. Members of this phylum have been associated with oral dysbiosis and inflammatory conditions [49] but have not been widely described in CKD. Given their low abundance and limited evidence in kidney disease, this finding should be interpreted cautiously and considered exploratory.

Network-based analyses revealed differences in microbial co-occurrence patterns between hemodialysis patients and controls, as well as across gender and dialysis vintage subgroups. These differences were reflected in network connectivity and clustering metrics. However, co-occurrence networks represent statistical associations rather than direct biological interactions and may be influenced by methodological factors such as sequencing depth and sample size. Therefore, these findings should be considered hypothesis-generating.

Functional predictions based on PICRUSt2 suggested differences in microbial metabolic potential associated with hemodialysis status, gender, and dialysis vintage. The predicted enrichment of pathways related to purine, carbohydrate, nucleic acid, and lipid metabolism in hemodialysis patients is consistent with previous CKD microbiome and multi-omics studies [50,51,52]. Gender-associated differences in predicted pathways further suggest that host-related factors may influence microbial functional potential, in agreement with recent metagenomic analyses in ESRD [50]. Functional predictions generated by PICRUSt2 should be interpreted cautiously because they represent inferred metabolic potential based on 16S rRNA gene profiles rather than directly measured metabolic activity. Experimental validation using metagenomic or metabolomic approaches will be required to confirm these predicted functional differences.

Dialysis vintage was also associated with differences in predicted functional profiles. Patients with shorter dialysis duration showed enrichment in pathways related to carbohydrate metabolism, whereas longer dialysis exposure was associated with increased representation of amino acid metabolism, stress-response pathways, and cell wall biosynthesis. These findings align with reports describing functional alterations across CKD severity stages [53,54,55] and may reflect microbial adaptation to prolonged exposure to the uremic environment.

Importantly, these functional inferences are based on predictive approaches and do not represent direct measurements of microbial activity. Further validation using metagenomic or metabolomic approaches is required to confirm these observations.

Overall, the functional prediction analysis supports the presence of dialysis-associated, gender-associated, and time-dependent differences in gut microbial functional potential, reinforcing the concept that hemodialysis is associated with complex and multifactorial alterations in the intestinal microbiome. These findings should be viewed as hypothesis-generating and provide a rationale for future multi-omics and longitudinal studies.

The present study has several limitations. First, the relatively small sample size limits statistical power, particularly for subgroup analyses according to sex and dialysis vintage, which should therefore be considered exploratory and hypothesis-generating. Second, residual confounding by clinical variables including diabetes mellitus, hepatitis C virus infection, inflammatory status, medication use (including antibiotics and phosphate binders), and dietary habits cannot be excluded. Although these variables were recorded, the relatively small cohort precluded reliable multivariable adjustment. Consequently, the observed associations between gut microbiota composition and hemodialysis should be interpreted with caution and considered hypothesis-generating pending validation in larger, well-controlled cohorts. Third, the functional predictions generated using PICRUSt2 are computational inferences and require experimental validation. Finally, validation in larger, multicenter cohorts is warranted.

Despite these limitations, this study provides a comprehensive descriptive analysis of the gut microbiota composition, interaction patterns, and inferred functional potential in hemodialysis patients, highlighting the relevance of dialysis vintage and gender as associated factors. These findings contribute to the growing body of evidence supporting the role of the gut microbiome in ESRD and underscore the need for larger, longitudinal, and multi-omics studies to clarify the clinical significance of microbiota alterations in patients undergoing hemodialysis.

## 5. Conclusions

This study provides a comprehensive characterization of gut microbiota alterations in patients with end-stage renal disease undergoing hemodialysis, highlighting its associations with dialysis treatment, vintage, and patient gender. Compared with healthy controls, patients undergoing hemodialysis exhibited distinct microbial community structures, reduced bacterial richness, altered co-occurrence patterns, and differences in predicted functional potential. Longer dialysis vintage appears to be associated with a greater magnitude of microbiota alterations, with the most pronounced differences observed after two years of hemodialysis.

In addition to taxonomic and ecological alterations, predicted functional profiling suggests that hemodialysis status and dialysis vintage are associated with distinct microbial metabolic potentials, particularly involving pathways related to carbohydrate utilization, nucleotide biosynthesis, amino acid metabolism, lipid metabolism, and cell wall–associated functions, highlighting the progressive and multifactorial nature of gut microbiota remodeling in patients undergoing hemodialysis. Ultimately, these insights aim to enhance the quality of life for patients with ESRD by informing personalized treatment plans that address microbiota imbalances. Such a patient-centered approach could lead to the development of targeted interventions that improve overall health outcomes, highlighting the potential of microbiome research to transform kidney disease management.

Given the cross-sectional design and limited cohort size, subgroup-specific observations should be interpreted with caution and confirmed in larger, independent cohorts. Nonetheless, these findings underscore the potential of gut microbiome profiling as a non-invasive strategy for characterizing gut microbiota shifts and exploring associations with maintenance hemodialysis, dialysis vintage, and gender. Future longitudinal studies incorporating metagenomics, metabolomics, and clinical outcome data are warranted to validate and extend these observations.

## Figures and Tables

**Table 1:** Average relative abundances of the most abundant bacterial genera present in fecal samples from 19 hemodialysis patients and 18 control subjects (mean relative abundance > 1%) in at least one study group (Figure 2A).

Genus	Control	HD
*Bifidobacterium*	1.718 ± 0.54	2.868 ± 0.63
[*Ruminococcus*] *torques* group	0.235 ± 0.07	5.367 ± 1.16
*Faecalibacterium*	7.428 ± 0.96	9.947 ± 1.35
*Prevotella* 9	22.731 ± 3.72	4.579 ± 1.23
*Bacteroides*	21.954 ± 2.63	8.55 ± 1.43

**Table 2:** Topological properties of networks in fecal communities of 18 healthy and 19 hemodialysis samples. HD: hemodialysis.

Groups	Control	HD
Number of nodes	226	259
Number of edges	1273	898
Number of positive interactions/copresence	498	448
Number of negative interactions/mutual exclusion	775	450
Clustering coefficient	0.245	0.201

**Table 3:** Mean relative abundances of the most abundant bacterial genera present in fecal specimens from patients undergoing hemodialysis over time and from healthy individuals: short period (<2 years; *n* = 7), long periods (≥2 years; *n* = 12), and 18 control subjects.

Bacterial Taxa	Control	Long	Short
*Bifidobacterium*	1.718 ± 0.55	2.032 ± 0.64	4.067 ± 0.61
[*Ruminococcus*] *torques* group	0.235 ± 0.07	3.363 ± 0.66	8.243 ± 1.6
*Lachnospiraceae* UCG-008	0.658 ± 0.09	5.573 ± 0.64	9.566 ± 1.88
*Roseburia*	2.641 ± 0.26	11.354 ± 2.18	5.328 ± 1.1
*Ruminococcus* 2	0.379 ± 0.14	14.113 ± 2.01	7.368 ± 1.66
*Faecalibacterium*	7.428 ± 0.97	11.738 ± 1.55	7.376 ± 1.01

## Data Availability

The original data presented in the study are openly available in the NCBI Sequence Read Archive (SRA) at BioProject PRJNA1490759 (Sample accessions: SAMN61437103–SAMN61437213).

## Supplementary Materials

The following supporting information can be downloaded at: https://www.mdpi.com/article/10.3390/microorganisms14081791/s1, Table S1: Demographic and clinical data of hemodialysis patients and healthy individuals using amplicon sequence analysis; Table S2: Detailed statistics for alpha and beta diversity metrics, and dispersion tests for the studied samples; Table S3: Sequencing quality metrics and read statistics of hemodialysis patients and healthy individuals; Table S4: Alpha-diversity indices for the studied sample groups. The mean values with the standard error (SE) are presented for Cohort, Gender (also by separating the control group; “extended”), and HD duration; Table S5: List of differentially abundant genera identified through multivariate analysis using MaAsLin3. Significant values are highlighted in blue (*p* ≤ 0.05, q ≤ 0.1). Bacterial genera are ordered from the smallest to the highest q-value; Table S6: Topological properties of the fecal communities’ networks in samples from female and male hemodialysis patients; Table S7: Topological properties of networks in fecal communities of samples from patients on hemodialysis according to the hemodialysis period; Table S8: Comparison of metabolic pathways predicted using PICRUSt2 between the control and HD groups, female and male patients, control and short dialysis period, and control and long dialysis period. Pathways with significantly different abundances (*p*-adj ≤ 0.05) are highlighted in blue and sorted from the smallest to the highest adjusted *p*-value; Table S9: Differentially abundant predicted metabolic pathways identified through multivariate analysis using MaAsLin3. Significant values are highlighted in blue (*p* ≤ 0.05, q ≤ 0.1); Figure S1: MultiQC graph of mean sequence quality (Q, Phred Score) of raw sequencing reads; Figure S2: Sequencing depth distribution for each sample of hemodialysis patients and healthy individuals; Figure S3: Differentially abundant bacterial genera identified through multivariate analysis using MaAsLin3. The forest plot shows beta coefficient values for the main factor (Cohort; control vs. HD patients). The control group is used as the reference. The heatmap contains the remaining covariates. Each covariate on the x axis is compared with its corresponding reference (Hemodialysis duration short, Diabetes yes, HTA yes, Gender M, Alcohol yes, ATB yes, Hepatitis C yes, and Sport yes); Figure S4: Alpha diversity metrics for gender after separating the control group into males (MC) and females (FC); Figure S5: Heatmap depicting all the significantly differentiated (q ≤ 0.05) MetaCyc pathways between control individuals and hemodialysis (HD) patients within the short-dialysis subgroup. Rows represent significant pathways clustered using Euclidean distance and complete linkage. Columns correspond to individual samples; Figure S6: Functional prediction using PICRUST2. Full list of differentially abundant pathway profiles (q ≤ 0.05) within the long-term subgroup. Rows represent significant pathways clustered using Euclidean distance and complete linkage. Columns correspond to individual samples; Figure S7: Differential abundance heatmap of PICRUSt2 predicted metabolic pathways that are significantly associated with clinical and lifestyle covariates (MaAsLin3). Only pathways passing FDR correction (q ≤ 0.1) for at least one covariate are displayed. Cells contain β coefficient values calculated from the MaAsLin3 model. Statistical significance is indicated by asterisks: * < 0.1 and ** for <0.01. Covariates include antibiotic exposure (ATB), Hepatitis C status, alcohol consumption, Gender, hypertension (HTA), cohort (HD vs. control), diabetes, hemodialysis duration (long vs. short), tobacco use, and sport activity. Each covariate value is compared with its corresponding reference value (ATB no, Hepatitis C no, Alcohol no, Gender M, HTA no, Cohort control, Diabetes no, Hemodialysis duration short, Tobacco no, and Sport no).
